# Cluster investigation report: a probable urine-associated transmission event of severe fever with thrombocytopenia syndrome—a case report

**DOI:** 10.3389/fpubh.2026.1905032

**Published:** 2026-10-06

**Authors:** Yan Su, An Chengcheng, Chen Qiongli, Li Zhiyuan, Li Shuwen, Pan Jingjing, Li Yi, Wang Haifeng, Huang Xueyong, Ye Ying

**Affiliations:** 1 Institute for Communicable Disease Control and Prevention, Henan Center for Disease Control and Prevention, Zhengzhou, China; 2 Henan Provincial Key Laboratory of Infectious Disease Pathogens, Zhengzhou, Henan, China

**Keywords:** case report, cluster event, human-to-human transmission, public health interventions, severe fever with thrombocytopenia syndrome, urine-associated transmission

## Abstract

**Introduction:**

Severe fever with thrombocytopenia syndrome (SFTS) is an emerging tick-borne viral disease with a high case fatality rate. Human-to-human transmission has been increasingly documented, primarily through direct contact with blood or bloody secretions. On June 21, 2025, a case of SFTS was reported in Zhumadian City, Henan Province. On July 3, a second case—epidemiologically linked to the first—was reported to the local Centers for Disease Control (CDC) through the notifiable disease reporting system, following laboratory confirmation on July 1.

**Case presentation:**

A 77-year-old female farmer with underlying comorbidities was hospitalized with fever, leukopenia, and thrombocytopenia, and subsequently died of SFTS. Her 31-year-old granddaughter, who had an open wound on her hand, cared for the patient and cleaned her urine. Eleven days later, she developed fever, nausea, vomiting, headache, fatigue, and myalgia. Laboratory tests confirmed SFTS virus infection by RT-PCR, with an initial cycle threshold (Ct) value of 38.85, which subsequently decreased to 31.48 over the following 2 days, indicating active viral replication. She recovered after antiviral therapy and was discharged on July 7. Comprehensive epidemiological investigation ruled out other potential transmission routes, including blood contact, tick bites, and environmental exposure. However, direct virological evidence from the index patient’s urine and genomic sequencing to confirm viral homology between the two cases were not available.

**Conclusion:**

This report describes a probable human-to-human SFTS transmission event associated with urine exposure through an open wound. While the absence of direct virological and genomic evidence precludes definitive confirmation, the convergence of epidemiological, clinical, and temporal evidence strongly supports this transmission route. This case highlights the need for enhanced protective measures—particularly the use of gloves when handling any body fluids, including urine—during care of SFTS patients.

## Introduction

Severe fever with thrombocytopenia syndrome (SFTS) is a newly discovered viral infectious disease in China in 2010, caused by the Dabie bandavirus (formerly SFTS virus) (1). The disease is characterized by fever, thrombocytopenia, leukopenia, and gastrointestinal symptoms, with severe cases often progressing to multi-organ dysfunction and hemorrhagic manifestations (2, 3). Nationwide surveillance data from 2010 to 2023 reported a total of 27,457 SFTS cases across 27 provinces in China, with the annual number of cases increasing from 71 in 2010 to 5,062 in 2023. The overall case fatality rate during this period was 4.82%, with annual fatality rates ranging from 2.70 to 12.70% (4, 5). However, the fatality rate varies considerably across studies and regions, with reported rates ranging from approximately 5 to 20% in overall estimates and as high as 30% in hospitalized patients or severe cases (1, 2). A global meta-analysis of studies published up to 2023 estimated a pooled case fatality rate of 7.80% (95% CI: 7.01–8.69%). Geographically, SFTS cases are predominantly reported in seven central-eastern provinces—Shandong (28.74%), Henan (22.89%), Anhui (20.83%), Hubei (14.34%), Liaoning (5.16%), Zhejiang (3.61%), and Jiangsu (3.49%)—which together account for 99.05% of all reported cases in China. The disease demonstrates a marked seasonal pattern, with most cases occurring from April to October and peaking from May to July. Middle-aged and older adult(s) farmers are the most affected population. Henan Province is one of the provinces with the highest incidence of SFTS in China.

The primary transmission route is tick bites, predominantly by *Haemaphysalis longicornis,* although human-to-human transmission through contact with blood or bloody secretions of infected patients has been increasingly documented (6). To date, urine has been recognized as a route of viral excretion in animal models and SFTS patients (7, 8), but human-to-human transmission via urine exposure has not been previously reported. This case report describes a probable urine-associated transmission event of SFTS between two family members in Zhumadian City, Henan Province, in June–July 2025.

## Case description

### Index case

A 77-year-old woman, a farmer living alone in A Township, A County, Zhumadian City, had a history of hypertension, coronary heart disease and emphysema. She underwent cataract surgery 2 months prior. Around June 13, she developed fever (peak 39.0 °C on June 13–14), accompanied by chills, general weakness and pain, occasional cough, wheezing and dyspnea after activity. On the afternoon of June 19, she was admitted to the People’s Hospital of A County, then transferred to ICU due to critical symptoms with decreased white blood cells and platelets. On the afternoon of June 20, she was transferred to the Central Hospital of Zhumadian City, and later that night, suspected of SFTS, she was transferred to No. 154 Hospital of Xinyang City. Until June 20, there was no significant bleeding or oozing of bloody secretions.

On the night of June 20, she was admitted to ICU and placed on a ventilator. Blood samples were collected upon admission on the evening of June 20; SFTS virus nucleic acid testing performed on June 21 returned positive with a CT value of 11.05. On June 21, her condition deteriorated sharply, and the family abandoned treatment. All auxiliary treatment tools were removed upon discharge. She died at home on the morning of June 22. A funeral was held at home from June 22 to June 23 noon. Family members arranged the remains, reporting no obvious bleeding or bloody secretion on the body surface. All participants wore masks and gloves. The case was cremated on June 23 and buried on June 24.

### Secondary case

A 31-year-old woman, granddaughter of the first case, was healthy with no underlying diseases. Since June 1, she had no history of tick bites, no exposure to tea plantations or wild fields, and no agricultural labor history. On June 18, she accidentally injured her left wrist while cooking and did not treat it.

On the morning of June 20, she went to care for the index case at the People’s Hospital of A County. During the entire care process, she had no direct contact with the patient’s blood or other body fluids, except for urine. Specifically, she cleaned the patient’s urine using a bedpan, and the open wound on her hand was contaminated by urine splashes. She felt a burning sensation in the wound when washing her hands afterward. She did not participate in any invasive procedures, nor did she handle the patient’s bloody secretions, as the patient had no obvious bleeding or oozing throughout the entire hospitalization. She then accompanied the index case during transfer to other hospitals but remained at the bedside without performing any medical or nursing tasks. After the index case returned home and died, the secondary case returned to A Township. During the funeral period, she did not participate in arranging or handling the remains. After the burial on June 24, she returned home and stayed indoors without outings until the onset of symptoms on July 1.

On July 1, she developed subjective fever at home, accompanied by nausea, vomiting, headache, fatigue, and myalgia, and was subsequently admitted to No. 154 Hospital of Xinyang City, where her temperature was 37.2 °C on arrival. Blood routine showed low white blood cells, and SFTS virus nucleic acid test was positive (Ct value: 38.85). She was hospitalized in the Department of Infectious Diseases and was cured and discharged on July 7. The case was formally reported to the local health authority on July 3 through the China Information System for Disease Control and Prevention.

### Laboratory findings

The specific test results for both cases are shown in Table 1. The index case had a very high viral load (Ct 11.05), while the secondary case had a lower viral load (Ct 38.85 at diagnosis). For the secondary case, the initial Ct value at diagnosis was 38.85, which fell within the manufacturer-defined ‘indeterminate’ range (38 < Ct ≤ 40). In accordance with the manufacturer’s instructions (The detailed manual has been sent as a Supplementary material 1), repeat nucleic acid extraction and RT-PCR testing were performed. The repeat testing yielded a consistent Ct value of 38.82, and the sample was therefore interpreted as positive for DBV nucleic acid according to the manufacturer’s criteria (repeat testing with Ct ≤ 40). The Ct value subsequently decreased to 33.03 on July 2 and 31.48 on July 3, demonstrating a clear trend of increasing viral load.

**Table 1:** Laboratory test results of cases in a SFTS cluster event.

Case classification	Sampling time	Sampling site	Blood routine test		DBV nucleic acid testing/Interpretation*	
			WBC	PLT	Result	CT value
First case	6/19	People’s Hospital of a County	1.92	53	/	/
	6/20	People’s Hospital of a County	7.68	43	/	/
	6/20	Central Hospital of Zhumadian City	10.71	47	/	/
	6/20	No. 154 Hospital of Xinyang City	11.9	47	/	/
	6/21	No. 154 Hospital of Xinyang City	15.2	61	+	11.05
	6/22	No. 154 Hospital of Xinyang City	24.69	199	+	15.78
Secondary case	7/1	No. 154 Hospital of Xinyang City	3.51	214	+	38.85
	7/2	No. 154 Hospital of Xinyang City	1.92	190	+	33.03
	7/3	No. 154 Hospital of Xinyang City	7.32	163	+	31.48
	7/4	No. 154 Hospital of Xinyang City	5.31	158	+	39.38

*According to the manufacturer’s instructions, samples with 38 < Ct≤40 are considered “indeterminate” and require repeat testing; if repeat testing yields Ct≤40, the sample is interpreted as positive. The secondary case’s initial Ct values (38.85 and 39.38) fell within this range; repeat testing yielded consistent results (38.82 and 39.38, respectively), and the samples were therefore interpreted as positive according to the manufacturer’s criteria. The “Interpretation” column reflects the final result based on this repeat testing protocol.

Notably, the July 4 sample showed a Ct value of 39.38 on initial testing (also within the indeterminate range), with repeat testing yielding 39.38, which was also interpreted as positive per the manufacturer’s criteria. However, as discussed below, this value was obtained after the patient had already shown clinical improvement following antiviral therapy, and likely reflects declining viral load rather than a false-positive result. The Ct value subsequently decreased to 33.03 on July 2 and 31.48 on July 3, demonstrating a clear trend of increasing viral load. Based on the combination of repeated testing, typical clinical manifestations, and the dynamic decrease in Ct values, the results were interpreted as positive for acute SFTS infection.

Importantly, the Ct value subsequently decreased to 33.03 on July 2 and 31.48 on July 3, demonstrating a clear trend of increasing viral load—a pattern consistent with active viral replication rather than non-specific amplification or contamination. Based on the combined assessment of (1) repeat testing results, (2) typical clinical manifestations of SFTS (fever, nausea, vomiting, headache, fatigue, myalgia, and leukopenia), (3) the dynamic decrease in Ct values over time, and (4) the clear epidemiological exposure history, the final clinical interpretation was positive for acute SFTS infection. It is important to distinguish between the RT-PCR assay result itself (which was near the detection limit) and the final clinical diagnosis, which was based on the synthesis of all available evidence.

### Environmental and tick surveillance

The village of the index case is in a plain area with many bushes next to the home. Cattle and sheep breeding are present locally. Emergency tick monitoring was carried out twice near the patient’s house; 4 parasitic ticks and 13 free ticks were captured, all testing negative for SFTS virus nucleic acid. Environmental samples from the wards of the People’s Hospital, Central Hospital, and No. 154 Hospital, as well as from the funeral site, all tested negative for SFTS virus nucleic acid.

### Screening of at-risk individuals

Five risk persons who participated in the care and funeral of the first case were identified. Blood samples were collected; all tested negative for SFTS virus nucleic acid, and IgM and IgG antibodies were negative by ELISA. After 2 weeks of population monitoring, no further human-to-human transmission occurred.

### Epidemiological conclusion

Combined with epidemiological investigation and laboratory examination, this event was determined to be a local cluster of cases most likely transmitted through urine, involving two infected persons. The index case was presumably infected by a tick bite during routine agricultural work. The secondary case was not properly protected during care and was most likely infected by contact with the patient’s urine. However, direct virological evidence from the index patient’s urine was not available, and the absence of viral genome sequencing means that alternative explanations—including independent infection of the secondary case through an unrecognized exposure—cannot be completely excluded.

## Discussion

This cluster occurred from late June to early July, which coincides with the peak epidemic season of SFTS in Henan Province. The secondary case in this cluster was likely infected on June 20, with the most probable route being contamination of an open hand wound by the first case’s urine. The first case had an extremely high viral load (CT value of 11.05), indicating massive viremia and suggesting that large quantities of infectious virus may have been present in various body fluids, including urine.

The strong seasonality of SFTS—with most cases occurring between April and October and peaking from May to July—is primarily driven by the activity patterns of its primary vector, the hard tick *Haemaphysalis longicornis*. The survival, development, and reproductive cycles of *H. longicornis* are significantly influenced by environmental conditions, particularly temperature and relative humidity. During late spring and early summer, rising temperatures and increased rainfall create optimal conditions for tick proliferation and host-seeking behavior. Multivariate modeling studies have identified temperature and relative humidity as independent risk factors for SFTS distribution (9). Additionally, seasonal agricultural activities—such as tea picking, farming, and forestry work—peak during this period, increasing human exposure to tick-infested habitats in hilly and mountainous areas (9). The combination of peak tick activity and heightened human outdoor activity during May to July thus creates optimal conditions for SFTS transmission, explaining the pronounced seasonal peak observed in Henan Province and other endemic regions.

Dabie bandavirus (DBV), a novel bunyavirus in the genus *Bandavirus* (family *Phenuiviridae*), causes systemic infection with multiorgan involvement. The virus can infect renal tissue, and acute kidney injury (AKI) is a well-recognized complication in severe SFTS cases (10). Viral shedding in urine likely results from direct viral replication in renal parenchymal cells or from filtration of circulating virus through the glomerular barrier during high-level viremia. A multicenter cohort study of 189 SFTS-confirmed patients demonstrated that DBV could be shed through urine in severely ill patients (11). Importantly, the excretion of DBV in urine was positively correlated with viral load in the blood. Animal models have provided further support: in ferrets infected with DBV, large amounts of infectious virus were shed through urine from 2 days post-infection and persisted until 12 days post-infection, with a longer shedding duration in urine compared with serum (12). These findings collectively establish that urine is a bona fide route of viral excretion in DBV-infected hosts.

The Ct value is inversely proportional to viral load—lower CT values indicate higher viral loads. In the present cluster, the index case had a blood Ct value of 11.05, representing an exceptionally high viremia. A large cohort study identified that urinary DBV levels are positively correlated with plasma viral load, and patients with urinary DBV levels greater than 6,379 copies/mL were at a higher risk of death within 28 days (10). In patients with SFTS-associated AKI, significantly higher viral loads were detected in both blood and urine compared with non-AKI patients. Importantly, urine viral loads can be substantial: in one study, patients with urinary DBV levels as high as 1.07 × 10^7^ to 3.23 × 10^7^ copies/mL were documented, with corresponding Ct values of 21.27 and 19.75. Notably, the presence of infectious virus in urine has been confirmed by successful isolation in Vero E6 cells from patient urine samples with Ct values below 35 (10). While urinary Ct values are generally higher (indicating lower viral loads) than blood Ct values in the same patient, the presence of infectious virus in urine—combined with the prolonged shedding period—makes urine a clinically relevant transmission vector (12). In the present case, the index patient’s blood Ct value of 11.05 suggests an extraordinarily high systemic viral burden, which likely resulted in substantial viral shedding in urine, consistent with the positive correlation between blood and urine viral loads documented in the literature.

On the other hand, previous studies have shown that human-to-human transmission of SFTS is predominantly associated with direct contact with patient blood or bloody secretions. A systematic review reported that human contact via exposure to bodily fluids significantly increased the odds of infection (13) (OR: 6.27, 95% CI: 1.23–42.81), and exposure to bloody secretions from corpses carried an even higher risk (14, 15). In this event, detailed epidemiological investigation ruled out the possibility of the secondary case’s exposure to blood or bloody secretions: the index case had no obvious bleeding or oozing of bloody secretions throughout the entire clinical course; the secondary case, aside from cleaning urine, did not participate in any procedures that might involve blood contact and did not participate in arranging the remains. Although DBV has been detected in urine in previous studies (7, 8), and animal experiments have suggested urine-mediated transmission, the evidence in our study remains circumstantial.

It’s worth noting that DBV transmission through broken skin is biologically and anatomically plausible. The skin serves as the primary portal of entry for DBV in natural tick-borne infection, where the virus is introduced through the tick’s mouthparts into the dermis. Skin-resident cells at the bite site—including immature Langerhans cells, epidermal dendritic cells, keratinocytes, and mast cells—are potential targets for DBV infection. Once the skin barrier is breached, the virus can access these susceptible cell populations. Open skin wounds bypass the intact stratum corneum, which serves as the body’s primary physical barrier against pathogen entry. Unlike intact skin, which is resistant to viral penetration, an open wound exposes the underlying dermis and provides direct access to susceptible cell types, including dermal fibroblasts, endothelial cells, and tissue-resident immune cells. This route of entry is mechanistically analogous to needle-stick injuries, which have been documented as a potential mode of DBV transmission in healthcare settings. Direct inoculation of virus-containing body fluids into the subcutaneous tissue through percutaneous exposure effectively bypasses multiple host defense barriers. Similarly, transmission through mucosal membranes (such as ocular exposure) has also been reported, but the skin wound route may be even more efficient because it provides direct access to the vascular and lymphatic systems without the need for viral penetration through epithelial layers. The presence of an open wound, combined with the high viral load in the index patient’s urine and the temporal association between exposure and symptom onset in the secondary case, provides a coherent mechanistic explanation for the probable transmission event in this cluster.

It should be noted that although the secondary case reported no history of tick bites, tick bites can often go unnoticed due to the anesthetic properties of tick saliva and the small size of ticks, especially in their larval and nymphal stages. Therefore, the absence of a reported tick bite history does not completely exclude the possibility of tick-borne transmission. However, several lines of evidence argue against this possibility: (1) the secondary case had no exposure to tea plantations, wild fields, or agricultural activities since June 1; (2) she lived in a plain area rather than the hilly or wooded environments typically associated with tick exposure; and (3) the close temporal and spatial relationship between her urine exposure and symptom onset strongly supports human-to-human transmission as the most plausible route.

In this cluster, the secondary case had an initial Ct value of 38.85 at diagnosis, which is near the assay’s detection threshold (Ct ≤ 38). This high Ct value indicates a low abundance of viral RNA in the peripheral blood at the time of sampling. Several factors may contribute to such borderline results, including the early stage of infection, low viral replication at the time of sampling, or degradation of nucleic acids during specimen transport and storage. Notably, the Ct value decreased to 33.03 on the following day (July 2) and further to 31.48 on July 3, demonstrating a clear downward trend consistent with active viral replication rather than non-infectious nucleic acid remnants or contamination. This temporal pattern of decreasing Ct values—indicating increasing viral load—supports the interpretation that the initial borderline-positive result represented genuine early-stage infection. Furthermore, the patient developed typical clinical manifestations of SFTS (fever, nausea, vomiting, headache, fatigue, and myalgia) and presented with leukopenia, both of which are consistent with acute SFTS infection. The combination of clinical symptoms, laboratory findings, and the dynamic change in Ct values over time provides strong supportive evidence that the secondary case represented a true infection rather than a false-positive result.

It is important to clarify the distinction between the RT-PCR assay result and the final clinical interpretation in this case. According to the manufacturer’s instructions for the RT-PCR kit used in this study (Beijing Zhuocheng Huisheng Biotechnology Co., Ltd.), a sample is considered positive if it shows a typical S-shaped amplification curve in the FAM channel with a Ct value ≤ 38. For samples with 38 < Ct value ≤ 40 in the FAM channel, provided that the VIC (HEX) channel shows adequate amplification (Ct ≤ 38), the result is interpreted as ‘indeterminate’ and repeat testing is required. If the repeat testing still shows a Ct value ≤ 40 in the FAM channel, the sample is considered positive. The secondary case’s initial Ct value of 38.85 therefore fell within the ‘indeterminate’ range, not the clearly positive range, but repeat testing yielded a consistent Ct value of 38.82, and the sample was therefore interpreted as positive per the manufacturer’s criteria. Similarly, the July 4 Ct value of 39.38 fell within the indeterminate range; repeat testing yielded a consistent value of 39.38, and the sample was interpreted as positive according to the same criteria. These results were further supported by the subsequent decreasing Ct trend and the patient’s clinical presentation. The secondary case’s initial Ct value of 38.85 and the July 4 value of 39.38 therefore exceeded the assay’s positivity threshold and would technically be considered negative by the assay alone. However, in clinical practice, laboratory results are always interpreted in the context of the patient’s overall clinical picture. The final diagnosis of SFTS in the secondary case was based on a synthesis of multiple lines of evidence: (1) a clear epidemiological exposure history (open wound contaminated with the index patient‘s urine, 11 days before symptom onset, consistent with the known incubation period of SFTS); (2) typical clinical manifestations (fever, nausea, vomiting, headache, fatigue, myalgia, and leukopenia); (3) serial Ct values showing a consistent decreasing trend from 38.85 to 33.03 to 31.48 over three consecutive days, indicating active viral replication; and (4) clinical recovery following antiviral therapy. The repeat testing of the initial sample (Ct 38.85) yielded consistent results, confirming the presence of low-abundance viral RNA. This case illustrates that while assay cutoffs provide important standardized criteria, clinical diagnosis in borderline situations requires integration of all available clinical, epidemiological, and laboratory data.

It should be noted that the Ct value on July 4 was 39.38, representing a transient increase compared with the previous day (31.48 on July 3). This fluctuation may be explained by several factors. First, the patient received antiviral therapy (ribavirin) and supportive care after admission, which may have suppressed viral replication and led to a decline in viral load. The transient increase in Ct value (i.e., decrease in viral RNA) likely reflects the therapeutic response and clinical improvement, consistent with the patient’s recovery and discharge on July 7. Second, as the Ct value approached the detection limit of the assay, random variation in RT-qPCR amplification efficiency may have contributed to the observed fluctuation. Third, differences in specimen collection and processing between sampling days cannot be excluded. Despite this single-day variation, the overall trend from July 1 to July 3—a consistent decrease in Ct values—supports the diagnosis of acute infection, and the subsequent increase in Ct value on July 4 is consistent with clinical recovery.

### Strengths and limitations

This case report has several strengths. The investigation was conducted through comprehensive epidemiological tracing, systematic screening of all at-risk individuals, and extensive environmental and vector surveillance. The detailed exposure history and the temporal relationship between exposure and symptom onset provide strong circumstantial evidence for the proposed transmission route.

However, several limitations must be acknowledged. Due to the delayed initiation of the cluster investigation, samples from the index case could not be obtained for sequencing and could not be compared with the secondary case to confirm viral homology. Without genomic confirmation, alternative explanations—including independent infection of the secondary case through an unrecognized tick bite or other exposure—cannot be completely excluded. Additionally, urine samples from the index case were not available for viral isolation or nucleic acid testing, which would have provided direct evidence of urinary viral shedding. Therefore, the most critical link in the proposed transmission chain—detection of viral RNA or infectious virus in the index patient’s urine—remains unverified. The evidence for urine-mediated transmission therefore remains circumstantial. Furthermore, while the decreasing Ct trend in the secondary case supports active infection, we did not perform viral culture to confirm the presence of infectious virus in the patient’s blood. The detection of viral RNA by RT-PCR does not necessarily equate to the presence of replication-competent virus.

Despite the absence of genomic confirmation, several lines of epidemiological evidence collectively support human-to-human transmission as the most plausible explanation for this cluster. First, the secondary case had no recognized exposure to tick-infested environments or agricultural activities during the 2 weeks prior to symptom onset, effectively ruling out the most common route of primary SFTS infection. Second, the only identified high-risk exposure was the contamination of an open wound with the index patient’s urine, which occurred 11 days before symptom onset—consistent with the known incubation period of SFTS (typically 7–14 days). Third, the index patient had an exceptionally high viral load (CT 11.05), making substantial viral shedding into body fluids highly likely. Finally, extensive environmental and tick surveillance, as well as screening of five other close contacts, yielded no evidence of alternative exposure sources. While genomic sequencing would have provided definitive confirmation, the convergence of epidemiological, clinical, and temporal evidence strongly supports the plausibility of the proposed transmission route.

### Takeaway lessons

This case report highlights that urine should be considered a potential vehicle for SFTS transmission in healthcare and home care settings. Health education and protective guidance should be strengthened for family members of SFTS patients during care and after treatment is withdrawn, particularly emphasizing the use of gloves when handling any body fluids, including urine, to reduce the occurrence of human-to-human transmission clusters.

## Conclusion

This report describes a probable human-to-human SFTS transmission event associated with urine exposure through an open wound and highlights the growing threat of SFTS. While definitive confirmation would require viral isolation from urine and genomic sequencing to confirm viral homology, the convergence of epidemiological, clinical, and temporal evidence strongly supports the plausibility of this transmission route. Enhanced protective measures—particularly the use of gloves when handling any body fluids, including urine—should be emphasized for family members and caregivers of SFTS patients.

## Data Availability

The raw data supporting the conclusions of this article will be made available by the authors, without undue reservation.
