# Supplementary material for: Cluster investigation report: a probable urine-associated transmission event of severe fever with thrombocytopenia syndrome—a case report
Source: Front Public Health. 2026 Oct 6;14:1905032. doi: 10.3389/fpubh.2026.1905032 (PMC13680459; doi:10.3389/fpubh.2026.1905032)
Supplement: Supplementary file 1 [file Supplementary_file_1.pdf]

Methods

Epidemiological investigation: Epidemiological methods were adopted to characterize the clinical and epidemiological features of the cases. A case definition was established, and contact tracing was performed to identify all individuals who had cared for or had close contact with the first case, including family members and funeral participants. A standardized questionnaire was used to collect information on demographics, clinical symptoms, exposure history (tick bites, outdoor activities), and potential routes of transmission.

Laboratory testing: All specimens were processed in a Biosafety Level 2 laboratory by trained personnel following standard operating procedures and quality control protocols. Qualitative detection of Dabie Banda virus (DBV) nucleic acid was performed using a Real-Time PCR Diagnostic Kit for Rapid Detection of Dabie Banda virus (TaqMan Probe-based) (Beijing Zhuocheng Huisheng Biotechnology Co., Ltd., Beijing, China; Catalog No.: A9041YH-50T, Lot No.: T202412151). The kit targets the conserved region of the M segment of DBV, with forward primer (M-F-3): 5'-AAGAAGTGGCTGTTCCATCATATTG-3', reverse primer (M-R-3): 5'-GCCTTAAGGACATTGGTGAGTA-3', and probe (M-Probe-3): 5'-FAM-TCATCCTCCTTGGATATGCAGGCCTCA-BHQ-2-3'. The reaction mixture (25 μL per tube) contained 18 μL of Nucleic Amplification Reaction Mix, 2 μL of Enzyme Mix, and 5 μL of RNA template, and was conducted on a Roche LightCycler 96 system under the following cycling conditions: 50°C for 10 min for reverse transcription, 95°C for 30 sec for initial denaturation, followed by 45 cycles of 95°C for 5 sec and 60°C for 30 sec for amplification and fluorescence acquisition. A sample was considered positive if it showed a typical S-shaped amplification curve in the FAM channel with a Ct value ≤38. For samples with 38<Ct value ≤40 in the FAM channel, provided that the VIC (HEX) channel showed a Ct value ≤38 (indicating adequate sample quality), the result was interpreted as ‘indeterminate’ according to the manufacturer's instructions, and nucleic acid extraction and RT-PCR were repeated in duplicate using the same RNA extract to confirm the result. If the repeat testing still showed a typical S-shaped amplification curve in the FAM channel with a Ct value ≤40, the sample was considered positive for DBV nucleic acid; otherwise, it was considered negative. Positive and negative controls were included in each run, and results were verified by two independent technicians. The limit of detection of this method was 5×10^2 copies/mL. Only samples that yielded consistent positive results in repeated testing were recorded as positive. It should be noted that this repeat testing protocol was a predefined institutional laboratory procedure for managing borderline results, rather than a post hoc decision made after reviewing the patient‘s clinical presentation. The purpose of this protocol is to reduce the risk of false-negative results in early-stage infections where viral RNA may be present at low abundance. Positive and negative controls were included in each run, and results were verified by two independent technicians. The limit of detection of this method was 5×10^2 copies/mL.

Antibody detection by ELISA. Levels of human IgG and IgM antibodies against DBV

were determined by indirect ELISA using commercially available kits (Severe Fever with Thrombocytopenia Syndrome Bunyavirus IgG/IgM Antibody Detection Kits, Suzhou Zhongke Sujing Biotechnology Co., Ltd., China). Serum samples were tested in duplicate following the manufacturer's instructions. A sample was considered positive if the optical density (OD) value was equal to or greater than the cutoff value (calculated as 0.10 plus the mean OD of the negative control wells), with negative control OD values below 0.05 treated as 0.05. Positive and negative controls were included on each plate, and results were verified by two independent technicians.

Environmental and vector surveillance: Ticks were collected near the patient's house (both parasitic ticks from domestic animals and free-living ticks from vegetation) and tested for SFTS virus nucleic acid. Environmental samples from the hospitals where the first case was treated were collected by local CDC staff.

Public health interventions: Health education on SFTS prevention and control was provided to at-risk individuals, and health monitoring was carried out. Terminal decontamination was performed after the cremation of the first case.

新型布尼亚病毒核酸

实时荧光 PCR 检测试剂盒说明书

【产品名称】

通用名称：新型布尼亚病毒核酸实时荧光 PCR 检测试剂盒（TaqMan 探针法）

英文名称：Real-Time PCR Diagnostic Kit for Rapid Detection of New Bunia virus

【产品货号】 A9041YH-25T、A9041YH-50T

【包装规格】 25 反应/盒、50 反应/盒

【预期用途】

本试剂盒适用于定性检测从全血或血清及其培养物提取的新型布尼亚病毒（大别班达病毒）核酸。实验结果仅为基础研究提供参考，不作为临床诊断依据。

新型布尼亚病毒（大别班达病毒）是 2010 年中国疾病预防控制中心分离并确认的一种新病毒。该病毒可引起发热伴血小板减少综合征。重症患者常表现为多脏器功能障碍甚至多脏器功能衰竭。

【储存条件及有效期】

1. 运输：试剂盒必须在冷冻条件下运输。
2. 保存：-20±5℃保存，应避免反复冻融。试剂盒冻融次数不超过 7 次。
3. 有效期：12 个月，请在有效期内使用。

【适用仪器】

经过多通道校正的全自动荧光PCR检测仪，需含FAM和VIC(HEX)检测通道。如ABI7500、7500FAST、Bio-Rad CFX96、Roche LightCycler 480II、上海宏石 SLAN-96P 等全自动荧光 PCR 检测仪。

【检验原理】

本试剂盒采用实时荧光 PCR 技术，适用于从全血或血清及其培养物中提取的新型布尼亚病毒（大别班达病毒）核酸检测。每一个反应体系中均含有检测新型布尼亚病毒（大别班达病毒）基因的特异性引物和荧光探针，通过收集 PCR 扩增产生的荧光信号，可对新型布尼亚病毒（大别班达病毒）核酸进行定性检测。

此外，每一个反应体系中均加入了针对人临床标本检测的内标对照的特异性引物和荧光探针，对待测样本的采集、运输和提取过程进行监控，提示检测结果的假阴性。

【主要组成成分】

序号	组分名称	25T 规格	50T 规格
1	核酸扩增反应液 Mix（A9041YH）	450μL×1 管	900μL×1 管
2	酶混合液	50μL×1 管	100μL×1 管
3	A9041YH-阳性对照	100μL×1 管	100μL×1 管
4	阴性对照	1000μL×1 管	1000μL×1 管

注意：不同批次试剂盒成分不可混用！

【样本要求】

从全血或血清及其培养物提取的 RNA 样品，均可使用本试剂盒进行检测。

血清：未加抗凝剂的血液自然凝固后，吸取上清液。

【操作步骤】

1. 样品制备

将全血或血清及其培养物按照病毒RNA提取试剂盒中相应的要求和步骤提取，推荐使用北京卓诚惠生生物科技股份有限公司的提取试剂（备案号：京昌械备20200008号或京昌械备20220148号），按试剂说明书进行核酸提取。提取的RNA可直接用于检测，若样本提取后不立即检测，也可存于-70℃备用，应避免反复冻融。

2. 反应体系配制

（1）体系配制：从试剂盒中取出试剂室温融化，待试剂完全解冻，颠倒混匀后瞬时离心。若待检样品数量为 n（n=样本数+阳性对照+阴性对照），则按 n+1 个反应配制体系。反应体系配制如下表。

试剂	1个反应体系的量	n+1个反应体系的量
核酸扩增反应液 Mix（A9041YH）	18μL	18μL×（n+1）
酶混合液	2μL	2μL×（n+1）

（2）体系分装：将上述反应液混匀离心后，按照每管20μL分装于荧光PCR仪适用的PCR管中。

（3）加样：分别取步骤1中提取的5μL RNA样品加入到分装好的PCR管中，扣紧管盖，轻弹混匀，瞬时离心后移至扩增区，总反应体积为25μL。阴性对照反应管中加入阴性对照5μL，阳性对照反应管中加入相应模板5μL。

3. 荧光 PCR 循环条件设置

<table>
<tr><th>程序</th><th>循环数</th><th>温度</th><th colspan="2">反应时间</th></tr>
<tr><td>1</td><td>1</td><td>50℃</td><td colspan="2">10min</td></tr>
<tr><td>2</td><td>1</td><td>95℃</td><td colspan="2">30sec</td></tr>
<tr><td rowspan="2">3</td><td rowspan="2">45</td><td>95℃</td><td colspan="2">5sec</td></tr>
<tr><td>60℃</td><td>30sec*</td><td>采集荧光</td></tr>
</table>

*其它仪器比如 ABI7500 采集荧光设定为 31sec，对结果无影响。

检测设置："Reporter Dye"设置为 FAM 和 VIC(HEX)，分别对应新型布尼亚病毒（大别班达病毒）核酸和内标对照的检测，"Quencher Dye"为 None。对于 ABI 系列仪器，注意将"Passive Reference"设置为 None。

4. 阈值设定

阈值设定原则为以刚好超过正常阴性对照的荧光信号最高点为阈值线，或根据仪器噪音情况进行调整。

5. 质控标准

阴性对照无扩增曲线，且阳性对照在两个检测通道均有 S 型扩增曲线，实验成立。否则视实验结果无效。

6. 结果分析与判定

a）若样品在 FAM 通道有 S 型扩增，且 Ct 值≤38，VIC(HEX)通道无要求，判定新型布尼亚病毒（大别班达病毒）核酸阳性；b）若样品在 FAM 通道有 S 型扩增，38＜Ct 值≤40，且 VIC(HEX)通道的 Ct 值≤38，判定为不确定样品，需重新提取核酸后进行检测；若复检的样品 FAM 通道仍有 S 型扩增，且 Ct 值≤40，则判定新型布尼亚病毒（大别班达病毒）核酸阳性，否则判定为阴性；c）若样品在 FAM 通道无明显 S 型扩增曲线，但报告有 Ct 值，且 VIC(HEX)通道 Ct 值≤38，仍判定为新型布尼亚病毒（大别班达病毒）核酸阴性；d）对于临床样本若样品在 FAM 检测通道无信号，且 VIC(HEX)通道 Ct 值＞38，请重新采集样本进行检测。

【检测方法的局限性】

本试剂盒检测的靶序列为新型布尼亚病毒（大别班达病毒）基因的保守区域，该靶基因高度保守稳定。但如果病毒在靶序列处发生基因突变，则可能出现假阴性结果，即发生漏检；同时，样品收集、处理、运送和保存的质量均对检测结果造成影响。

【产品性能指标】

1. 最低检出限：5×10^{2}copies/mL。
2. 线性范围：5×10^{2}~2×10^{10}copies/mL。
3. 交叉反应：针对可能与新型布尼亚病毒（大别班达病毒）产生交叉的其他病原（汉坦病毒、登革热病毒、基孔肯亚病毒、寨卡病毒、黄热病毒、裂谷热病毒、克里米亚-刚果出血热病毒、埃博拉病毒、鼠疫耶尔森氏菌、溶血性猪链球菌、立克次体、钩端螺旋体）均无交叉反应。
4. 精密度：检测精密度参考品的变异系数＜5%。

【注意事项】

1. 实验前请仔细阅读本试剂盒说明书，严格按操作步骤执行。
2. 试剂盒内各组分在使用前应充分融化混匀并经高速短暂离心后使用。
3. 试剂盒须避光保存，以免荧光物质衰减。所使用的离心管、Tip 头应高压灭菌，且不含 DNase 和 RNase。
4. 整个操作过程和 PCR 实验室的软硬件设施应符合卫生部颁布《医疗机构临床基因扩增检验实验室管理办法》、《医疗机构临床基因扩增检验实验室工作导则》等法规的要求。并恰当处理实验过程中产生的废物和扩增产物，防止交叉污染。

【生产企业】

企业名称：北京卓诚惠生生物科技股份有限公司

联系地址：北京市昌平区科学园路 37 号院 1 号楼正旦国际 C 座

电话号码：400-6500-984

公司网址：www.x-abt.com

【说明书版本信息】

版本编号：SMS-A9041YH-02L

批准日期：2025 年 4 月 8 日
